# Electrochemotherapy of colorectal liver metastases - an observational study of its effects on the electrocardiogram

**DOI:** 10.1186/1475-925X-14-S3-S5

**Published:** 2015-08-27

**Authors:** Barbara Mali, Vojka Gorjup, Ibrahim Edhemovic, Erik Brecelj, Maja Cemazar, Gregor Sersa, Branka Strazisar, Damijan Miklavcic, Tomaz Jarm

**Affiliations:** 1 University of Ljubljana, Faculty of Electrical Engineering, Trzaska cesta 25, SI-1000 Ljubljana, Slovenia; 2 University Medical Center Ljubljana, Zaloska cesta 2, SI-1000 Ljubljana, Slovenia; 3 Institute of Oncology Ljubljana, Zaloska cesta 2, SI-1000 Ljubljana, Slovenia

**Keywords:** Electroporation, electrochemotherapy, high voltage electric pulses, colorectal metastases, liver, electrocardiogram, post-operative care, side effects

## Abstract

**Background:**

Electrochemotherapy (ECT) is a combined treatment in which high voltage electroporation (EP) pulses are used to facilitate the uptake of a chemotherapeutic drug into tumor cells, thus increasing antitumor effectiveness of the drug. The effect of ECT of deep-seated tumors located close to the heart on functioning of the heart has not been previously investigated. In this study, we investigate the effects of intra-abdominal ECT of colorectal liver metastases on functioning of the heart during the early post-operative care period.

**Methods:**

For ECT high voltage EP pulses with amplitudes of up to 3000 V and 30 A were delivered in synchronization with electrical activity of the heart. Holter electrocardiographic (ECG) signals were obtained from 10 patients with colorectal liver metastases treated with ECT. ECG was recorded during the periods of 24 hours before and after the surgical procedure involving ECT. Four-hour long night-time ECG segments from both periods exhibiting the highest level of signal stationarity were analyzed and compared. Changes in several ECG and heart rate variability (HRV) parameters were evaluated.

**Results:**

No major heart rhythm changes (i.e., induction of extrasystoles, ventricular tachycardia or fibrillation) or pathological morphological changes (i.e., ST segment changes) indicating myocardial ischemia were found. However, we found several minor statistically significant but clinically irrelevant changes in HRV parameters after ECT procedures: a decrease in median values of the mean NN interval, a decrease in the low-frequency and in the normalized low-frequency component, and an increase in the normalized high-frequency component.

**Conclusions:**

Only minor effects of intra-abdominal ECT treatment on functioning of the heart were found. They were expressed as statistically significant but clinically irrelevant changes in heart rate and long-term HRV parameters and were as such not life-threatening to the patients. The nature of these changes is such that they can be attributed to the known effects of the drugs given to the patients in the post-operative care. Further investigation is still warranted to unambiguously resolve whether ECT with high voltage EP pulses applied in immediate vicinity of the heart is responsible for the observed effects.

## Background

Electroporation-based techniques, like electrochemotherapy (ECT), gene electrotransfection and non-thermal irreversible electroporation, are becoming a viable option for treatment of internal, deep-seated tumors and tissues (e.g., in bones, brain, liver, lung, prostate, kidney, colon and esophagus). In such cases, surgical, percutaneous or endoscopic procedures are used to gain access to the treatment area [1-18]. These advanced treatment procedures thus provide new possibilities for restoring, correcting or modifying physiological functions but, at the same time, because of their novelty, complexity and technical specificity, may also introduce new risks to patients. One of potential new risks is that application of electroporation (EP), i.e. high voltage electric pulses could interfere with functioning of the heart. In particular, in treatment of deep-seated tumors, the electric current can propagate through a larger volume of tissue surrounding the treated region due to absence of a protective barrier of the skin, relatively large inter-electrode distances, and high electric conductivity of internal tissues and organs, which is even further increased due to EP itself [19]. Therefore, an increased probability of EP pulses affecting the cardiac muscle and thus potentially causing heart-related effects exists in ECT treatment of deep-seated tumors located close to the heart (e.g. in liver, lung or esophagus) with respect to the same treatment applied to cutaneous or subcutaneous tumors. If the electric field distribution is such that the heart lies in the reversibly electroporated area, premature action potentials are generated causing cardiac arrhythmias [8].

During the study of intra-abdominal ECT of colorectal liver metastases at the Institute of Oncology Ljubljana, Slovenia [5,6] tumors located relatively close to the heart and surrounded with highly conductive tissue were treated with electric pulses with voltage and current amplitudes of up to 3000 V and 50 A respectively. Pulses were delivered synchronized with the ECG. Needle electrodes were inserted into liver tissue during open surgery at inter-electrode distances of up to 3 cm. Such conditions lead to increased probability of interaction of EP pulses with functioning of the heart. Indeed, according to published studies on non-thermal irreversible EP, where EP pulses with the same characteristics are applied as in ECT of colorectal liver metastases (although more pulses are used than in ECT), different effects on functioning of the heart were observed. Minor and major hemodynamic changes were reported, as well as cardiologic changes due to unsynchronized irreversible EP pulse delivery, such as systolic hypertension, supraventricular tachycardia, ventricular tachycardia with pressure drops, ventricular fibrillation, ST segment elevation and changes in T wave morphology [7,8]. In these studies, major cardiologic changes (ventricular arrhythmias) were terminated by defibrillation, whereas minor effects in general subsided after EP pulse delivery. However, even when delivery of irreversible EP pulses was synchronized with the absolute refractory period of the heart cycle, minor cardiac arrhythmias could still be induced [8]. The authors also reported that the ST segment elevation appearing due to myocardial ablation with synchronized irreversible EP pulses gradually disappeared but the situation did not normalize completely during the 45 min observation period after the treatment. Nevertheless, no myocardial injury was observed [8]. The use of ECG synchronization during IRE treatment has thus been greatly explored in the literature and has become a standard for clinical IRE therapy when treating tumors in close proximity to the heart [7,8,10,20-22].

During the clinical study on safety and feasibility of ECT of colorectal liver metastases at the Institute of Oncology Ljubljana we experienced no obvious or clinically relevant immediate or short-term effects of EP pulses on functioning of the heart during the treatment itself. Nevertheless, the observations summarized in the previous paragraph lead to the hypothesis that EP pulse delivery in close vicinity of the heart even when synchronized with ECG could induce measurable effects on functioning of the heart that could remain visible several hours after the treatment. The aim of this study was thus to explore these effects by evaluating changes in ECG during the early post-operative care of patients within 24 hours after intra-abdominal surgery and ECT of colorectal liver metastases.

## Methods

### Patients and the study

The study of changes in ECG of patients during early post-operative care after intra-abdominal electrochemotherapy (ECT) procedure was part of the prospective pilot clinical study conducted at the Institute of Oncology in Ljubljana (EudraCT number: 2008-008290-54; http://ClinicalTrials.gov: NCT01264952). The objective of this study was to examine safety and feasibility of ECT of colorectal liver metastases [5,6] with 16 patients included in the study.

Ambulatory 9-lead ECG records (I, II, III, aVR, aVL, aVF, V1, V3, V6) of approximately 24-hours duration were recorded on the day before and after intra-abdominal ECT procedure. ECG signals were sampled at 200 Hz and acquired using a holter system (SpiderView, ELA Medical, France). These ECG signals were used to investigate changes in functioning of the heart in early post-operative care. Holter recording before and after ECT was not possible for patients number 1, 2 and 8 and the signals recorded on patients number 3, 11 and 16 could not be used due to technical reasons. ECG signals from ten patients were thus included in the analysis. The main characteristics of these ten patients and their tumors are presented in Table 1; further patients' details can be found in [6]. The patients recruited in the study had no serious pre-existing cardiac condition.

**Table 1:** Main characteristics of the patients, tumors and EP pulse delivery for individual patient and tumor.

PATIENT				TUMOR		ELECTROPORATION PULSE DELIVERY						
**No**.^a^	**Sex**	**Age**	**Pre-existing cardiac conditions**	**Metastasis**	**Two largest perpendicular diameters (cm)**^b^	**Electrode geometry, length of active part**	**No. of electrodes used**	**No. of EP pulses delivered**	**Average voltage applied (V)**	**Average current applied (A)**	**Energy applied (J)**	**Total energy applied to patient (J)**

**4**	M	56	none	M1 M2	1.0 × 1.0 2.0 × 1.0	variable, 3 cm	5 6	65 72	1695 1875	16.69 19.67	196.1 275.1	471.2
**5**	M	54	none	M1 M2 M3	2.0 × 2.0 1.0 × 1.0 0.7 × 0.7	variable, 3 cm	6 5 4	110 64 53	2810 2131 2024	29.72 21.89 31.21	867.2 314.4 386.3	1567.9
**6**	M	69	hypertension, mild mitral regurgitation	M1 M2	3.5 × 2.5 1.5 × 1.5	fixed, 3 cm	7 7	672 96	713 713	6.67 6.30	321.8 47.4	369.2
**7**	M	59	none	M1 M2	1.0 × 1.0 1.5 × 1.2	variable, 3 cm	5 5	64 64	1052 1100	10.58 11.74	75.7 89.6	165.3
**9**	F	38	none	M1 M2	1.0 × 1.0 1.0 × 1.0	variable, 3 cm	5 5	64 64	1778 1778	26.82 24.16	312.1 266.4	578.5
**10**	M	69	hypertension	M1 M2	1.7 × 1.0 1.0 × 1.0	variable, 3 cm	5 5	64 64	1924 1744	24.40 17.25	304.7 177.7	482.4
**12**	F	57	hypertension	M1 M2	2.5 × 2.5 1.8 × 1.1	fixed, 3 cm	7 7	288 384	713 713	9.02 8.24	184.4 22.0	406.4
**13**	M	63	hypertension	M1	3.0 × 2.5	variable, 3 cm	5	75	2421	46.13	5976.0	5976.0
**14**	M	61	hypertension	M1 M2	1.0 × 1.0 1.0 × 1.0	fixed, 3 cm	7 7	96 288	718 718	4.59 3.77	32.0 81.5	113.5
**15**	F	62	hypertension	M1 M2	3.5 × 3.0 3.0 × 3.0	variable, 3 cm	6 6	104 111	2533 -	31.50 -	802.6 -	1605.2^c^

^a ^Patient numbers according to Edhemovic et al. [6], where more patients' details can be found. For patients not included in this study see the explanation in the Methods section.

^b ^The two longest perpendicular tumor diameters were estimated using ultrasound imaging during intra-abdominal surgery.

^c ^The data for voltage and current in tumor M2 in patient No. 15 was corrupted; therefore the total energy applied to the patient was estimated as twice the energy applied to tumor M1 since almost the same parameters of EP pulse delivery were used for both tumors.

### Electrochemotherapy procedure

The protocol of the clinical study was described in detail elsewhere [5,6]. Here it is summarized briefly and with emphasis on details pertinent to the investigation of the effects of ECT on ECG.

ECT procedures were performed under combined general and thoracic epidural anesthesia (TEA). Epidural catheter was placed at T8-12 interspaces before anesthesia, and a test dose of lidocaine (60 mg) was injected. 30 minutes before surgical incision, all patients received the prophylactic antibiotics cefazolin (2 g) and metronidazole (500 mg) as a rapid intravenous infusion. The patients received 25-50mg of levobupivacaine epidurally during the operation, according to analgesic needs. General anesthesia was induced with sufentanyl, propofol, and vecuronium, and after tracheal intubation, maintained with sevoflurane in 40% oxygen-nitrous oxide balance. A bolus of local anesthetic levobupivacaine (between 10 to 15 mg) was administered epidurally about five minutes before surgical incision. Additional epidural levobupivacaine and intravenous vecuronium and sufentanyl were administered during the operation as appropriate. Epidural analgesia (continuous epidural infusion of 0.25% levobupivacaine) with rate 3-6 mL/hour and with patient controlled analgesia (PCA) demand dose of 4 ml (lock out time 30 minutes) was continued in all patients postoperatively. During post-operative care, patients also received intravenously opioid analgesic piritramide, non-opioid analgesic metamizole and antiemetic metoclopramide as required. In the case of hypotension, an infusion of vasopressor norepinephrine in low dose (range from 0.01 - 0.2 µg/kg/min) was administered in order to maintain optimal blood pressure. At night, patients received another dose of the analgesic metamizole and the antipsychotic haloperidol.

ECT was performed during open surgery that was primarily performed to treat larger, unresectable or difficult-to-treat liver metastases [6]. To reach the tumors, two types of needle electrodes were used: individual needle electrodes for variable geometry positioning (VG1240, IGEA, Italy) with either a 3 or 4 cm long active (conductive) part and a diameter of 1.2 mm; and electrodes with fixed hexagonal geometry (N-30-HG, IGEA, Italy) with a 3 cm long active part, an electrode diameter of 0.7 mm and a fixed distance between the outer electrodes of 1.7 cm. The decision about selection of variable or fixed geometry electrodes depended on the tumor location and size. Four to six electrodes with variable geometry were used for larger tumors and tumors located deep inside the liver, whereas the electrodes with fixed hexagonal geometry were used for tumors located on the liver surface. When electrodes for variable geometry were used, a patient-specific treatment plan was prepared before the ECT procedure based on abdominal cross-sectional CT or MRI images. Treatment planning took into account the size and location of individual tumors and major blood vessels in order to define the number and geometrical distribution of the electrodes, distances between the electrodes, pairs of electrodes for EP pulse delivery, and voltages of EP pulses for each pair of the electrodes [2,5,6,23-25]. These procedures were described in detail in [24,25] and are available at http://www.visifield.com.

ECT procedure was performed as follows. First, needle electrodes were inserted into the tumor and the immediate surrounding tissue according to the treatment plan. The chemotherapeutic drug bleomycin was then administered intravenously in bolus with a dose of 15 mg/m^2^. Delivery of EP pulses was started eight minutes after bleomycin administration. Eight EP pulses of 100 μs duration were delivered for each pair of the electrodes by the electric pulse generator Cliniporator Vitae (IGEA, Carpi, Italy).

The delivery of EP pulses was synchronized with ECG via AccuSync 42, an external R wave triggering device (AccuSync, USA). The ECG signal was acquired by the AccuSync 42 independently of the regular ECG monitoring by the anesthesiologist. The AccuSync 42 detects the R wave of each individual heartbeat early on the ascending slope of the R wave and provides a trigger to Cliniporator Vitae. Cliniporator Vitae delivers pulses 50 ms after a valid trigger pulse, thus avoiding the so-called vulnerable period of the ventricles (the T-wave). Validation of trigger pulses is performed by a built-in synchronization algorithm.

The exact protocol of EP pulse delivery depended on the type of electrodes (for more details see also Table 1). In all cases 8 EP pulses with 100 θs duration were delivered for each electrode pair. Patients number 4, 7, 9, 10 and 15 were treated using electrodes for variable geometry. EP pulses were delivered in trains of 8 pulses with one EP pulse per valid trigger pulse. Patient number 13 was also treated using electrodes for variable geometry, the pulses however were delivered in subgroups of 4 pulses per valid trigger pulse at a repetition frequency of 1 kHz and with EP pulse polarity reversal between the two subgroups. Patients number 6, 12 and 14 were treated using electrodes with fixed hexagonal geometry. EP pulses were delivered in subgroups of 4 pulses per valid trigger pulse at a repetition frequency of 5 kHz and with EP pulse polarity reversal between the two subgroups. For variable electrode geometry the total number of pulses delivered to a tumor as well as voltage amplitudes depended on the treatment plan. For fixed electrode geometry on the other hand the total number of pulses (96 pulses per application) as well as the voltage amplitude (730 V) was fixed. See Table 1 for more details.

### ECG analysis

Analysis was performed on ECG signals recorded continuously over periods of 24 hours and was based on comparison of parameters derived from comparable periods before and after ECT procedure. The night-time interval between 0:00 and 4:00 a.m. was selected for analysis because activity of patients was found to be minimal during this period and therefore the corresponding ECG signals were relatively stationary. ECT was performed during daytime, usually before 12:00 a.m. so the two extracted parts of ECG signals for every patient were separated by 24 hours.

In order to detect and classify heartbeats, the initial processing of extracted four-hour long ECG signals was performed using a previously developed algorithm, which is described in detail elsewhere [26,27]. Briefly, this algorithm is an extended QRS detector which provides the Q, R and T peak locations and classifies individual heartbeats as either normal or abnormal (i.e., abnormal ECG shape or heart rhythm). For proper functioning of this algorithm, an ECG lead with high R wave amplitude and large dynamics within QRS complex in comparison to other parts of ECG signal are required. ECG leads I, II and V6 fulfil these requirements and were analyzed for all patients. For each patient individually, the ECG lead resulting in the highest number of detected heartbeats and the lowest number of abnormal heartbeats was selected as the most appropriate for further analysis. The number of ST segments deviating from the normal level was identified from the selected ECG lead using commercial holter analysis software (SyneScope, ELA Medical, France).

For reliable analysis of heart rate variability (HRV), noise-free 5 min long subsegments with a small number of abnormal heartbeats are required [28,29]. The threshold value for acceptable proportion of abnormal heartbeats for HRV analysis was initially chosen to be 1%. Each four-hour long ECG signal was divided into twelve 20-min long segments and one 5-min subsegment was extracted from each 20-min segment for HRV analysis. Each 20-min segment was scanned from its beginning for the first occurrence of a 5-min subsegment with less than 1% of abnormal heartbeats that at the same time started at least 10 minutes after the end of the 5-min subsegment from the preceding 20-min segment. A 5-min subsegment fulfilling both requirements was extracted and used for HRV analysis. If no 5-min subsegment from a given 20-min segment could be extracted at the 1% threshold value for abnormal heartbeats, the threshold value was gradually increased in increments of 1% (up to 5%) and the selection procedure was repeated until a suitable 5-min subsegment was found. The upper limit of 5% for the percentage of acceptable abnormal heartbeats was adopted from [30] where the authors analyzed the effects of editing abnormal heartbeats on calculation of HRV measures.

The whole procedure thus resulted in twelve selected 5-min segments for each ECG record that were appropriate for HRV analysis. They were additionally manually inspected for correctness of R peak detection, location and heartbeat classification and, if necessary, corrected to assure correct identification and classification of every single heartbeat. We developed software (using Matlab, MathWorks, USA) with graphical user interface for easy and rapid screening and checking for correctness of the located feature points and classification of heartbeats [31]. After screening and editing of R peak data, the locations of Q and T peaks were also verified (and corrected if necessary) but only for heartbeats classified as normal since the locations of Q and T peaks of abnormal heartbeats were unreliable and thus inappropriate for the analysis.

### Assessment of QT intervals

QT interval is usually defined as the interval between the start of a Q wave and the end of a T wave within an individual normal heartbeat. However, we used an interval between the Q peak and the T peak (peak-to-peak QT intervals, denoted as ppQT), because Q and T peaks are much easier to detect reliably than the start of a Q wave and the end of a T wave. The corrected ppQT intervals (denoted as ppQTc intervals) were determined for each heartbeat as the ppQT interval divided by the square root of the corresponding RR interval, following the common definition of the corrected QT interval.

### Assessment of heart rate variability

Heart rate variability (HRV) analysis deals with fluctuations in RR interval time series between normal adjacent QRS complexes. Normal QRS complexes are those resulting from the sinoatrial node depolarization, and the associated RR intervals are frequently referred to as NN intervals [28]. The RR interval series obtained from regular ambulatory ECG recordings are usually not directly suitable for HRV analysis because of inclusion of abnormal RR intervals, which is known to affect the results of HRV analysis in general and of frequency-domain HRV analysis in particular [28]. Therefore, additional preprocessing of RR interval series was needed in order to improve reliability of HRV analysis.

After manual editing of RR intervals from each selected 5-min subsegment of the ECG signals in the previous step, 1% or less abnormal heartbeats in each 5-min subsegment were required in order to minimize the error in subsequent calculation of HRV parameters. If this requirement was not met initially for a given subsegment, it was replaced with a new one, i.e., with the first next 5-min subsegment from the same 20 minutes long segment according to the requirements for selection and extraction of ECG subsegments as described above.

In the next step the 5-min subsegments were edited using cubic spline interpolation method to replace abnormal RR intervals with the interpolated RR intervals. This approach is considered superior to simple deletion of abnormal RR intervals [32]. An NN interval sequence of only normal RR intervals was thus obtained. The NN interval sequence was of course an irregularly sampled sequence (sampling intervals not of the same length). For frequency domain HRV analysis it was converted to a corresponding regularly sampled sequence. Cubic spline interpolation method at 1 kHz sampling rate was used to fit the non-equidistant NN sequence data. The resulting curve was then downsampled to 4 Hz sampling rate [27]. This equidistantly sampled NN sequence was finally used for frequency domain HRV analysis.

Following the recommendations in the literature for short-term ECG recordings [28], several commonly used linear HRV measures in time domain (mean NN, SDNN, SDSD, RMSSD, pNN50) and frequency domain (LF, nLF, HF, nHF, LF/HF), as well as nonlinear measures (Poincaré diagram descriptors, SD1, SD2 and SD1/SD2) were calculated from the NN interval sequences for each 5-min segment included in the analysis (for details see Table 2). Linear detrending of NN interval sequences was performed before calculation of HRV measures except for the mean NN interval and Poincaré descriptors to remove the low frequency baseline trend component. HRV measures were calculated for all twelve 5-min segments of each ECG signal. The median value for each HRV measure was calculated over all patients, separately for ECG signals recorded before and after ECT procedure. The comparison of these median values was then used for evaluation of the effects of ECT procedure on functioning of the heart. All calculations were performed in Matlab using our own tool for ECG and HRV analysis developed previously [31].

**Table 2:** Time-domain linear and nonlinear and frequency-domain HRV measures used in this study.

Parameter	Unit	Description
		***Time-domain measures***

**mean NN**	ms	Mean of normal-to-normal (NN) intervals
**SDNN**	ms	Standard deviation of NN intervals; an estimate of overall HRV
**SDSD**	ms	Standard deviation of differences between adjacent NN intervals; an estimate of short-term HRV; associated with parasympathetic activity
**RMSSD**	ms	Square root of the mean of the sum of the squares of differences between adjacent NN intervals; an estimate of short-term HRV; associated with parasympathetic activity
**pNN50**	%	Number of pairs of adjacent NN intervals differing by more than 50 ms divided by the total number of all NN intervals

		***Nonlinear measures***

**SD1**	ms	Standard deviation of the Poincaré plot perpendicular to the line-of-identity; the shortest diameter of the fitted ellipse; an estimate of short-term HRV; associated with parasympathetic activity
**SD2**	ms	Standard deviation of the Poincaré plot along the line-of-identity; the longest diameter of the fitted ellipse; an estimate of long-term HRV; associated with sympathetic activity
**SD1/SD2**	-	Ratio of SD1 to SD2; describes the ratio of short-term to long-term HRV; describes the relationship between parasympathetic and sympathetic activity

		***Frequency-domain measures***

**LF**	ms^2^	Power in low frequency range (0.04-0.15 Hz); estimate of long-term HRV; reflects both sympathetic and parasympathetic activity
**nLF**	n.u.	Normalized power of LF; LF/(LF+HF)x100; estimate of long-term HRV; reflects both sympathetic and parasympathetic activity
**HF**	ms^2^	Power in high frequency range (0.15-0.4 Hz); estimate of short-term HRV; reflects parasympathetic (vagal) activity
**nHF**	n.u.	Normalized power of HF; HF/(LF+HF)x100; estimate of short-term HRV; reflects parasympathetic activity
**LF/HF**	-	Ratio between LF and HF range powers; describes the relationship between sympathetic and parasympathetic activity, i.e. sympathovagal balance

Frequency-domain HRV measures were initially calculated from power density spectra estimated with non-parametric FFT-based Welch's periodogram method using Hamming window and with parametric autoregressive model-based method (AR model of the order 16 with coefficients determined based on Burg method) [28,29]. Since both approaches resulted in practically indistinguishable results, only the results of autoregressive model-based calculation of frequency-domain HRV measures are reported.

### Assessment of parameters of EP pulse delivery

The time course of voltage and current during EP pulse delivery was recorded and stored for further analysis. These records allowed us to determine the number of EP pulses delivered to each tumor and to calculate the average values of voltage and current and the total energy applied to individual tumor and patient. The median value of voltage and current delivered within each train of eight EP pulses was first calculated. Then, the median of these median values for all trains of EP pulses delivered to individual tumor was used as a measure of the average voltage and current delivered to the tumor. Where more than one tumor was treated in a patient, the largest average voltage and current values were taken for statistical analysis. The energy applied to individual tumors was determined by integrating the power of all EP pulses (i.e. product of current and voltage) delivered to the tumor. The total energy applied to a patient was also determined as the sum of energy delivered to all tumors in the patient. Values of these electrical parameters were intended for assessment of their association with the significant changes in ECG or HRV parameters (if found).

### Statistical analysis

Several statistical comparisons were performed. The median values of number of abnormal heartbeats, number of deviated ST segments, ppQTc intervals and HRV measures obtained from 4-hour ECG signals recorded before and after ECT procedure on individual patients were compared using paired Wilcoxon Signed Rank test. Linear regression method was used to determine the degree of association between the parameters of EP pulses and the ECG and HRV measures for which statistically significant changes were found. The statistical analysis was performed using SigmaPlot 11.0 package (Systat Software Inc., USA) and the p value of less than 0.05 was considered as the indication of statistically significant differences.

## Results

No clinically significant adverse effects such as induction of extrasystoles, ventricular tachycardia or fibrillation) on functioning of the heart in patients due to ECT procedure were observed in the post-operative care. The analysis of ECG signals recorded before and after ECT procedure showed no increase in appearance of abnormal heartbeats in ECG signals recorded after ECT even in cases where abnormal heartbeats were present in ECG signal before ECT (Table 3). No statistically significant difference in percentage of abnormal heartbeats and in percentage of deviated ST segments was found comparing periods before and after ECT procedure (p = 0.054 and p = 0.322, respectively, Table 3).

**Table 3:** Analyzed ECG leads and heartbeat intervals.

Patient number	ECG lead used for analysis before/after ECT	Analyzed heartbeat intervals	No. of beats before ECT procedure			No. of beats after ECT procedure		
			
			N	A (%)	ST (%)	N	A (%)	ST (%)
**4**	II / II	NN, ppQTc	14317	12 (0.084)	0 (0)	14216	17 (0.119)	0 (0)
**5**	II / II	NN, ppQTc	15722	3 (0.019)	15692 (99.8)	18112	1 (0.006)	14001 (77.3)
**6**	II / II	NN	13975	309 (2.163)	0 (0)	18271	8 (0.044)	0 (0)
**7**	II / II	NN, ppQTc	16873	20 (0.118)	0 (0)	18820	15 (0.080)	2321 (12.3)
**9**	II / V_6_	NN	18321	18 (0.098)	6915 (37.7)	26047	9 (0.035)	0 (0)
**10**	V_6 _/ I	NN	14785	70 (0.471)	0 (0)	15202	23 (0.151)	763 (5.02)
**12**	V_6 _/ V_6_	NN, ppQTc	18825	42 (0.223)	0 (0)	17764	6 (0.034)	4395 (24.7)
**13**	II / II	NN, ppQTc	13864	6 (0.043)	0 (0)	19405	8 (0.044)	3209 (16.5)
**14**	V_6 _/ V_6_	NN, ppQTc	13857	135 (0.965)	10111 (73.0)	16620	0 (0)	7078 (42.6)
**15**	II / II	NN, ppQTc	17461	25 (0.143)	696 (3.99)	26450	39 (0.147)	25188 (95.3)

**Summary**			158000	640 (0.403)	33414 (21.1)	190907	126 (0.034)	56955 (29.8)

NN: normal-to-normal heartbeat interval; ppQTc: corrected peak-to-peak QT interval; N: number of heartbeats classified as normal; A: number of heartbeats classified as abnormal; ST: number of heartbeats with ST segment deviation.

ECG signals recorded in patients number 6, 9 and 10 contained large amounts of high-frequency noise either before or after ECT procedure, which rendered a reliable determination of ppQTc intervals impossible (Table 3). On the remaining seven patients, the comparison of median values of ppQTc interval before and after intra-abdominal ECT indicated no statistically significant difference (p = 0.469, Table 4).

**Table 4:** Median changes in ECG and HRV parameters after ECT.

Evaluated parameters	Median change	Percentile		*p*
			
		25%	75%	
**mean ppQTc [ms]**	-5.03	-17.40	2.45	0.469
**mean NN [ms]**	-161.00	-260.66	-25.05	**0.020**
**SDNN [ms]**	-11.05	-18.09	-3.12	0.131
**SDSD [ms]**	-3.86	-11.26	0.272	0.232
**RMSSD [ms]**	-3.85	-11.24	0.275	0.232
**pNN50 [%]**	-0.18	-1.50	0.00	0.461
**SD1 [ms]**	-2.73	-7.96	0.19	0.232
**SD2 [ms]**	-15.95	-26.43	-3.52	0.065
**SD1/SD2**	0.01	-0.11	0.12	0.695
**LF [ms^2^]**	-93.15	-303.13	-30.15	**0.049**
**HF [ms^2^]**	-9.93	-49.76	38.86	0.625
**nLF [n.u.]**	-12.56	-16.81	-10.49	**0.049**
**nHF [n.u.]**	12.56	10.49	16.81	**0.049**
**LF/HF**	-1.04	-3.26	-0.90	0.432

Definitions of parameters are given in Table 2. Statistical significance of the change is presented in the right-most column (Wilcoxon signed rank test).

**Table 5:** Correlation between changes in the ECG and HRV parameters and the EP pulse delivery parameters.

Compared parameters	No. of delivered EP pulses	Average U applied to tumors	Average I applied to tumors	Total energy applied to patient
**Δ mean NN**	0.919	0.399	0.210	0.154
**Δ LF**	**0.026**	0.315	0.522	0.953
**Δ nLF**	0.239	0.616	0.271	0.060
**Δ nHF**	0.239	0.616	0.271	0.060

Correlation was evaluated for ECG and HRV parameters with statistically significant change after ECT from Table 4. Values of *p *(statistical significance of the correlation) are given in the table.

ECG signals from all 10 patients were included in HRV analysis. The Wilcoxon Signed Rank test showed statistically significant decrease in median values of parameters mean NN interval, LF and nLF, and statistically significant increase in median nHF after intra-abdominal ECT (Table 4). Statistically significant negative linear correlation was found only between changes in the parameter LF and the number of EP pulses applied during EP pulse delivery (p = 0.026; Table 5).

## Discussion

The results of our study indicate that intra-abdominal surgery involving ECT of colorectal liver metastases was associated with only minor and clinically irrelevant changes in functioning of the heart detected in early post-operative care and as such presented no life threat for the patients. No major changes in functioning of the heart such as rhythm changes (i.e., increased induction of abnormal heartbeats, ventricular tachycardia or fibrillation) or pathological morphological changes (e.g., ST segment changes) that would suggest myocardial ischemia were found in ECG signals.

According to the literature, surgery and anesthesia produce a stress response characterized by increased sympathetic and hormonal activity which may predispose the patient to arrhythmias [30]. Post-operative arrhythmias are thus quite common and affect about 7% of patients that underwent major non-cardiothoracic surgery. Post-operative arrhythmias occur more commonly in older patients, most often in the first four days after surgery and are frequently associated with other underlying complications [33-35]. In addition, EP pulses alone delivered to tissue located near the heart can induce abnormal heartbeats, even if EP pulses are synchronized with the refractory period of the cardiac cycle [7,8,36]. The cardiotoxicity of bleomycin, the chemotherapeutic drug used in intra-abdominal ECT, can also provoke changes in ECG often manifested as an appearance of or an increase in the incidence of premature atrial contractions, or as appearance of supraventricular tachycardia, sinus bradycardia or other conduction abnormalities [37]. However, the abnormal heartbeats caused by bleomycin are of transient nature, occurring during and shortly after the drug administration, and would be expected to disappear by the night time period for which the effects of ECT procedure on ECG were evaluated in our study. The frequency of abnormal heartbeats was not increased after ECT procedure (Table 3), which suggests that ECT procedure on its own did not induce additional post-operative arrhythmias.

EP pulses delivered in immediate vicinity of the heart could potentially cause structural changes in myocardium and consequently induce myocardial ischemia. The myocardial ischemia can be detected by analysis of changes in ST segment level, by blood test of values of biomarkers of myocardial necrosis (such as cTn, CK-MB) and by cardiac imaging [38]. Minor or early stage of myocardial ischemia can be detected with blood tests, whereas major or late stage myocardial ischemia can be detected also from ECG. Changes in ST segment level were clearly documented after cardioversion and defibrillation [39-45], and were recognized to be a transient phenomenon (lasting between a few seconds to 24 hours) and not associated with structural changes of myocardium. Our results show no statistically significant change in proportion of heartbeats with changes in ST segment level in ECG signals recorded after ECT procedure. We can thus conclude that ECT procedure had no effects resulting in ST segment changes that would suggest the presence of major myocardial ischemia. In the future blood tests of cardiac biomarkers could be included in evaluations to investigate potential early stage myocardial ischemia caused by EP pulses.

Heart rate variability (HRV) analysis is a method for studying the physiological mechanisms responsible for the control of heart rate, in which the autonomic nervous system appears to play the primary role. HRV measures strongly depend on conditions under which ECG signals are recorded and are affected by numerous physiological factors modulating the normal rhythm of the heart (such as age and gender of the patient, blood pressure, drugs taken, presence of heart diseases, diabetes, renal failure, surgical procedure, pain, stress, smoking, alcohol consumption, exercise and sleep) [28,29,46]. When HRV measures are derived from stable ECG signals recorded under controlled, resting conditions, it is suggested that HRV measures are reliable metrics that can provide information of the degree of autonomic modulation of the heart [47].

There are many factors that can directly or indirectly affect the autonomic nervous system. In addition, any factors that change properties of the heart itself can also have an effect on the heart rate [48]. Even though the direct effects of EP pulses used in ECT on the autonomic nervous system were not expected, we performed HRV analysis on the recorded signals to evaluate changes in the parameters of HRV induced by the entire procedure, and to see if there were any changes not consistent with the known effects of either post-surgical stress response or the drugs (especially anesthetics and analgesics) used in postoperative treatment.

In our study, ECG signals recorded in the same night-time window when patients were resting (from 0:00 to 4:00 a.m.) were chosen for evaluation of HRV before and after ECT procedure in order to exclude the influence of circadian variation and patient's activity on HRV. Because HRV measures were compared for each patient before and after ECT procedure (self-control), age and gender dependence of HRV was minimized. None of the patients had clinically significant pre-existing cardiac conditions (Table 1) - more detailed patient information can be found in Edhemovic et al. [6]. As far as the ECT procedure was concerned, all patients received the same type of treatment: the same type of anesthesia and analgesia were used during surgery; the same type of surgical intervention was performed; similar duration of the procedure (between 6 and 8 hours); ECT was performed with intravenously administered bleomycin; and patients were exposed to similar pre- and post-operative care. HRV analysis showed statistically significant change in median values of three HRV parameters (Table 4). These effects could be attributed either to the effect of anesthesia, ECT itself, post-operative drugs, and post-operative stress and pain response or to a combination of these factors.

Induction and maintenance of general anesthesia with sevoflurane and N_2_O are known to cause a decrease in both the LF and HF components of the HRV power spectra [49-54]. The degree of change tends to depend on the concentration of sevoflurane being used [51]. The recovery period of HRV after general anesthesia with sevoflurane was reported to be short (from 30 to 180 minutes) [50,53,55]. The time interval between the end of surgical procedure and the time period from which the ECG record was taken for analysis was about 10 hours. It is therefore reasonable to assume that the effects of inhalation anesthesia on HRV had been eliminated by then.

Delivery of EP pulses could also contribute to an increased heart rate but such effects are reported to be only transient and were thus not expected to be present during the night-time after ECT procedure [56]. Interestingly, our results showed an unexplained statistically significant negative correlation between changes in the LF component of HRV and the number of EP pulses delivered to the patients (Table 5).

Epidural anesthesia decreases blood pressure and may be accompanied by a decrease in heart rate. The principal mode of action of epidural blockade is prevention of sympathetic outflow [57]. High thoracic epidural anesthesia (TEA) on level T1-T5 blocks the cardiac afferent and efferent sympathetic fibers with loss of chronotropic and inotropic drive to the myocardium [58]. In our patients epidural catheter was inserted at T8-T12. TEA at this level has been shown to have several benefits, including improved recovery after major abdominal surgery [59]. Epidural anesthesia restricted to the level of the low thoracic and lumbar region (T5-L4) results in a peripheral sympathetic blockade with vascular dilatation in the pelvis and lower limbs. Licker and his colleagues found that immediately after the operation with high TEA the total HRV and both LF the HF components (as well as LF/HF ratio) were significantly decreased with only the HF component returning to nearly pre-operation level within less than 24 hours [60]. Similarly, Jideus et al. found that the HRV variable expressing sympathetic activity was significantly lower and that the postoperative increase in heart rate was less pronounced in the high-TEA group than in the control group after surgery [61]. In our study low TEA was used and thus we achieved splanchnic sympathetic block, the intention was therefore not to influence cardiac sympathetic fibers directly. However, some cranial spread of local anesthetic almost certainly occurred, especially because we used low concentration of levobubivacaine with relative high volumes of injectate. It has been found that low TEA (at T8/9) has indeed considerable cranial spread and that this spread is even more extensive in the elderly and with higher volumes of injectate [62]. In addition, our low TEA also resulted in sympathetic block of adrenal catecholamine release. All this means that our observations of the decrease in the LF component and lack of decrease in the HF component of HRV 10-12 hours after the operation were consistent with the known effects of thoracic epidural anesthesia and analgesia.

To avoid hypotension we used vassopresor norepinephrine at low infusion rates (0.01 - 0.2 µg/kg/min) in postoperative care. Norepinephrine vascular effects in the doses normally used clinically result from the simultaneous stimulation of alpha and beta adrenergic receptors in the heart and vascular system. This results in an increase in the force (and in the absence of vagal inhibition, in the rate) of myocardial contraction. Intravenous administration of norepinephrine results in intense vasoconstriction and results in increase arterial pressure. The increase in blood pressure may cause a reflex decrease in the heart rate [63,64]. Schächinger et al. found in their study on healthy volunteers a significant decrease in the LF component of HRV (they did not evaluate the HF component) and in the heart rate with norepinephrine infusion at 0.02-0.1 µg/kg/min [65]. Tulppo et al on the other hand found in their study on healthy volunteers a significant increase in the HF component of HRV, no change in LF component and a decrease in the heart rate with norepinephrine infusions at 0.05-0.15 µg/kg/min [66]. In our study the LF component of HRV was decreased, which is in agreement with the results of Schächinger et al., but the heart rate was increased. The decrease of the heart rate that could be expected as a reflex reaction to norepinephrine infusion was most likely offset by the complex post-surgical stress response in our patients. In fact, tachycardia is one of many typical symptoms of the stress response encountered in early postoperative intensive care [67].

According to the literature, analgesics given in post-operative care, especially opioids, depress the LF component more than the HF component of the HRV spectrum, and therefore reduce the LF/HF ratio, suggesting an increased parasympathetic dominance in the HRV activity 529 [68]. Therefore, the observed significant decrease of LF and nLF component (estimates of long-term HRV) after ECT procedure in our study were most likely due to several analgesics (piritramide, sufentanil, metamizole) and local anesthetics administered to patients in post-operative care.

Surgical pain and post-operative pain is known to trigger autonomic nervous and endocrine responses, which can be manifested also as accelerated heart rate [56,69]. Consequently, shortened NN intervals and elevation of the LF component were detected, but SDNN, HF, and total power of HRV were not found to be affected by the pain [56,69,70]. In our study, statistically significant decrease in mean NN interval after ECT procedure was detected but no elevation of the LF component was found, therefore, the heart rate was probably not increased due to post-operative pain.

## Conclusions

Intra-abdominal surgery involving ECT of colorectal liver metastases with synchronized delivery of EP pulses does not present any life threat for the patient. Namely, no major changes in functioning of the heart such as rhythm changes (i.e., increased induction of abnormal heartbeats, ventricular tachycardia or fibrillation) or pathological morphological changes (e.g., changes in ST segment level) due to ECT procedure were found in ECG signals recorded during early post-operative care. The probability for complications could increase when using EP pulses of longer durations or larger number of pulses of increased pulse repetition frequency and/or in the treatment of tumors in the immediate vicinity of the heart. Statistically significant although clinically irrelevant differences in some HRV measures after ECT procedure were identified but they can be explained by the known effects of post-operative drugs. In order to distinguish the effects of ECT itself from those of post-operative drugs, the ECG recordings before and after a similar surgical procedure but without ECT would be required, preferably obtained from the same patients on which the ECT has been performed during a previous surgical procedure. However, cases where a surgical procedure involving intra-abdominal ECT is followed by a similar surgical procedure without ECT are rare and were not available for this study. In addition, for reliable investigation of influence of different parameters of EP pulse delivery (such as number of delivered EP pulses, voltage, current and energy), a detailed data about distance between location of EP pulse delivery and the heart would also be needed.

## List of abbreviations

ECT: electrochemotherapy

EP: electroporation

ECG: electrocardiogram

HRV: heart rate variability

TEA: thoracic epidural anesthesia

PCA : patient controlled analgesia

## Competing interests

D. Miklavcic holds patents of which some have been licensed to IGEA SpA, Italy, the producer of a clinical device for electrochemotherapy used in this study. He also consults for IGEA SpA. Other authors declare no conflict of interest.

## Authors' contributions

BM collected and analyzed the ECG data and drafted the manuscript. VG was cardiology consultant. IE and EB were the main surgeons performing ECT in the study. MC helped to draft the paper. GS coordinated clinical parts of the study. BS was anesthesiology consultant. DM conceived the study and helped to draft and finalize the manuscript. TJ supervised and helped in collection and analysis of ECG signals and manuscript drafting and finalized the manuscript. All authors read and approved the final version of the manuscript.

## Declarations

Publication costs for this article were funded by the Research Agency of the Republic of Slovenia.

This article has been published as part of *BioMedical Engineering OnLine *Volume 14 Supplement 3, 2015: Select articles from the 6th European Conference of the International Federation for Medical and Biological Engineering (MBEC 2014). The full contents of the supplement are available online at http://www.biomedical-engineering-online.com/supplements/14/S3.
